# Age–Period–Cohort Analysis of Trends in Mortality from Drowning in China: Data from the Global Burden of Disease Study 2015

**DOI:** 10.1038/s41598-018-24281-7

**Published:** 2018-04-11

**Authors:** Zhenkun Wang, Chuanhua Yu, Henry Xiang, Gang Li, Songbo Hu, Jinhui Tang

**Affiliations:** 1 Medical Department, Tongji Hospital, Tongji Medical College, Huazhong University of Science and Technology, Wuhan, 430030 China; 2 School of Health Sciences, Wuhan University, Wuhan, 430071 China; 3 Center for Injury Research and Policy & Center for Pediatric Trauma Research, The Research Institute at Nationwide Children’s Hospital, The Ohio State University, Columbus, OH 43210 USA; 4 College of Medicine, The Ohio State University, Columbus, OH 43210 USA; 5 Department of Paediatrics, Tongji Hospital, Tongji Medical College, Huazhong University of Science and Technology, Wuhan, 430030 China; 6 Global Health Institute, Wuhan University, Wuhan, 430071 China

## Abstract

The studies on drowning mortality are very scarce in China, and the aim of this study is to identify the long-term patterns of drowning mortality in China between 1990 and 2015 to provide evidence for further prevention and control on drowning. The mortality data were derived from the Global Burden of Disease Study 2015 and were analyzed with the age–period–cohort framework. This study demonstrated that the age-standardized mortality rates for drowning in both sexes displayed general declining trends with a decrease in the drowning mortality rate for every age group. In the same birth cohort, both sexes witnessed a substantial decline followed by a slight increase in the risk of death from drowning with age after controlling for period deviations. The estimated period and cohort relative risks were found in similar monotonic downward patterns for both sexes, with more reduction for females than for males during the whole study period.

## Introduction

Drowning remains an important public health problem around the world. As indicated by the World Health Organization, it is one of the 10 leading causes of death for individuals aged 1–24 years in every region of the world, and the 3rd leading cause of unintentional injury death globally, representing 7% of all injury-related deaths^1^. In 2013 alone, drowning caused around 368 100 deaths^2^ and 21 608 000 disability-adjusted life years^3^ worldwide. Over 90% of all the drowning fatalities occurred in low- and middle-income countries^4^; however, related researches and prevention strategies have been largely confined to high-income countries, especially the epidemiological studies on drowning mortality at the national level.

It has been shown that China ranked the highest for drowning deaths and had the second highest drowning mortality rate in the world^5^. However, the studies on drowning mortality are very scarce in China. Of existing literature in English, there were only integrated injury studies with a few drowning mortality figures on certain time points^2^, rather than the short-term trend studies on drowning mortality in China. Existing studies in Chinese on drowning mortality did include some trend analyses, but their research scope had been confined to a specific region or population with a relatively short or out-of-date study period; in addition, there has been no comprehensive analysis on the underlying reasons for those temporal trends. To address these limitations, this study aims to describe and investigate the long-term trends of drowning mortality from 1990 to 2015 in China, examining and assessing the influence of age-, period-, and cohort-specific effects by sex under the age–period–cohort (APC) framework, using data from the Global Burden of Disease Study 2015 (GBD 2015). Discoveries from this study could give hints on resource allocation focusing the vulnerable for the prevention of drowning, as well as enhance the understanding of mortality trends and provide certain etiologic implications of drowning in China.

## Methods

### Data sources

Data used in this study were derived from the GBD 2015, which has provided internally consistent estimates of age-sex specific all-cause and cause-specific mortality for 240 causes of death globally, regionally and nationally for 1990–2015^2^. There were five main data sources GBD 2015 adopted to provide data on causes of death in China, and the data of mortality of drowning were mainly extracted from three of them, i.e. Disease Surveillance Points, Maternal and Child Surveillance System and Chinese Center for Disease Control and Prevention Cause of Death Reporting System^6^. Causes of death were identified based on the 9th and 10th revision of the International Classification of Disease. Drowning deaths and corresponding population information were obtained for the subsequent statistical analyses. To describe the temporal trends, drowning mortality rates for males and females in China were age-standardized by the GBD global age-standard population^6^. This study did not involve any interaction with human subjects or personal identifying information, so research ethics committee approval and patient consent were not necessary in this case.

### Statistical analysis

The aim of APC analysis is to assess the contributions of age, period and cohort effects on the outcome such as demographic or disease rates. The age effects represent a differing risk of the outcome associated with different age brackets; the period effects represent variations in the outcome over time that influence all age groups simultaneously; the cohort effects are associated with changes of the outcome across groups of individuals with the same birth years^7,8^. Holford has proposed that^9^ if age, period, and cohort trends are orthogonally decomposed into their linear and nonlinear parts, many useful functions^10–15^ can be estimated. In this study, we mainly focused on the following estimable functions^16^. Net drift, the overall log-linear trend by calendar period and birth cohort, indicates the overall annual percentage change; local drifts, the log-linear trend by calendar period and birth cohort for each age group, indicate annual percentage changes for each age group; longitudinal age curve indicates the fitted longitudinal age-specific rates in reference cohort adjusted for period deviations; the period (or cohort) RR would be the period (or cohort) relative risk adjusted for age and non-linear cohort (or period) effects in a period (or cohort) versus the reference one^8,16^.

To conduct APC analysis, the mortality and population data of drowning were arranged into consecutive 5-year periods from 1990 to 2015 and successive 5-year age intervals from 0–4 years to 75–79 years (individuals over 80 were not considered in this study since they were only recorded as one group in GBD database). We obtained the estimable parameters by the Age-period-cohort Web Tool^16^ (Biostatistics Branch, National Cancer Institute, Bethesda, MD, USA). The central age group, period, and birth cohort were defined as the reference respectively in all age-period-cohort analyses; in case of an even number of categories, the reference value was set as the lower of the two central values^16^. Wald Chi-Square tests were adopted for the significance of the estimable functions. All statistical tests were two-sided, and P < 0.05 was considered statistically significant.

## Results

**Figure 1:**
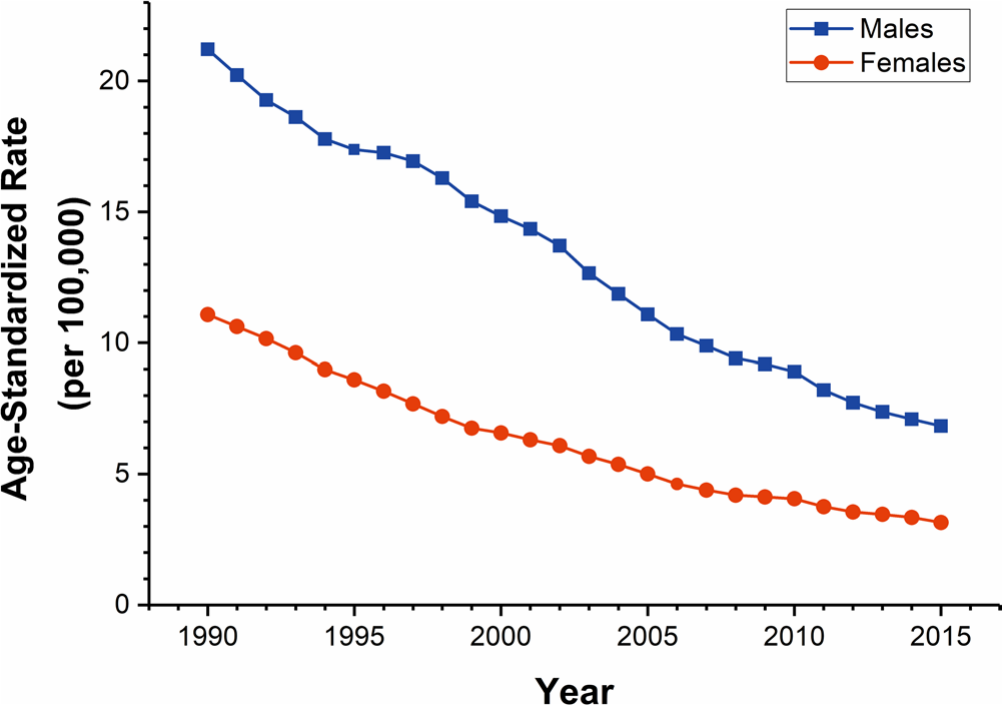
Trends of the age-standardized mortality rates (using GBD global age-standard population) per 100,000 population for drowning by sex in China, 1990–2015.

**Figure 2:**
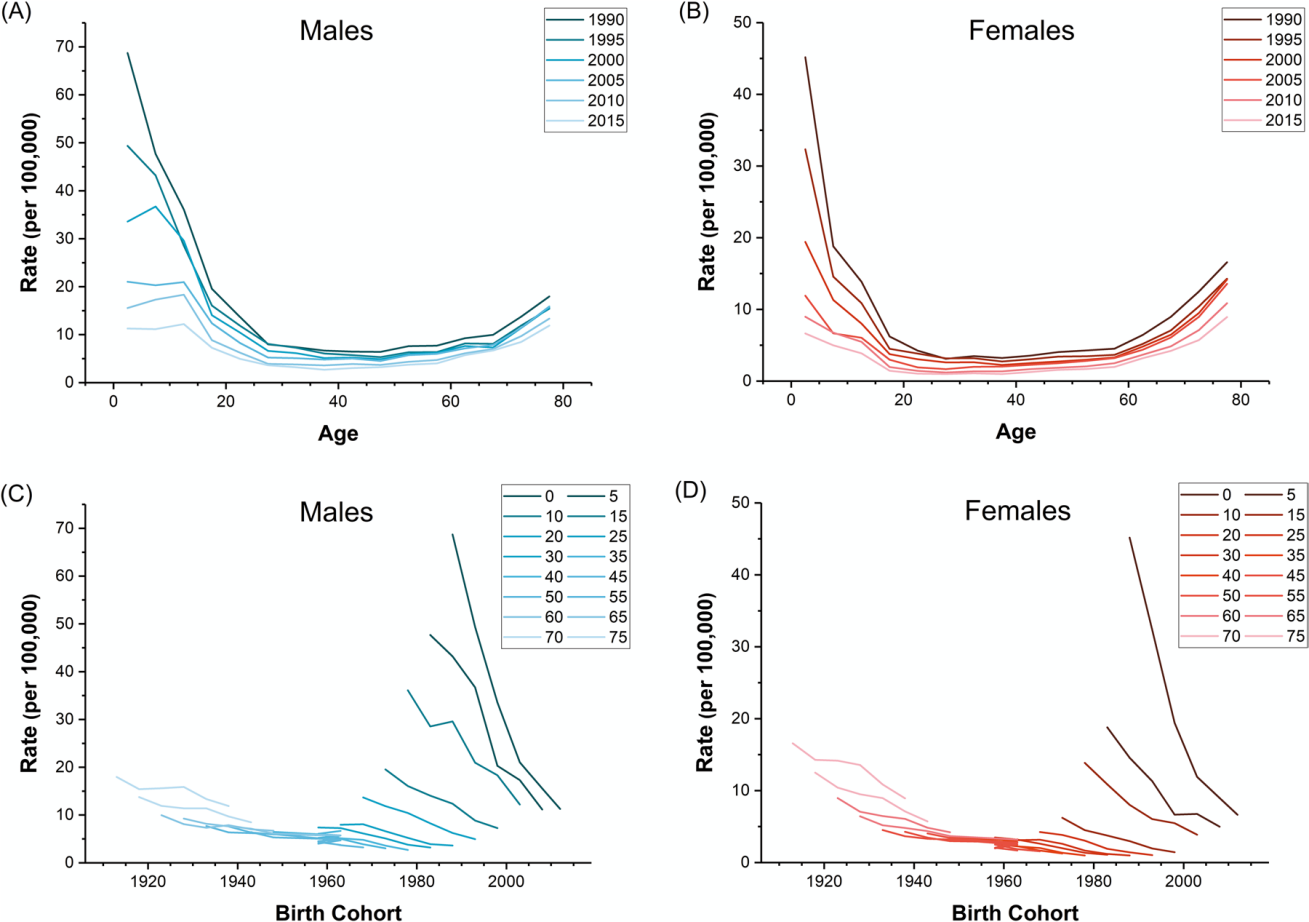
Age-specific mortality rates of drowning by period of death and cohort-specific mortality rates of drowning by age group, stratified by sex during the period of 1990–2015. (**A**–**B**) In the first row represent the age-specific mortality; (**C**–**D**) in the second row represent the cohort-specific mortality.

Overall, a total of 2,162,565 and 916,767 deaths from drowning were reported among 17,223,535,373 and 16,287,671,499 person-years at risk for males and females during 1990–2015 in China, respectively. It was shown that the age-standardized mortality rates (ASMR) for drowning in both sexes displayed constant declining trends, from 21.21 to 6.83 per 100,000 for males and 11.08 to 3.15 per 100,000 for females (Fig. 1). The reduction in the ASMR for drowning from 1990 to 2015 was 67.80% in males and 71.57% in females. The variations in the age-specific mortality rates for drowning among Chinese males and females between 1990 and 2015 indicated the existence of a period effect (Fig. 2A,B), and the non-parallelism among the age curves by birth cohort suggested the existence of a cohort effect (Fig. 2C,D).

**Figure 3:**
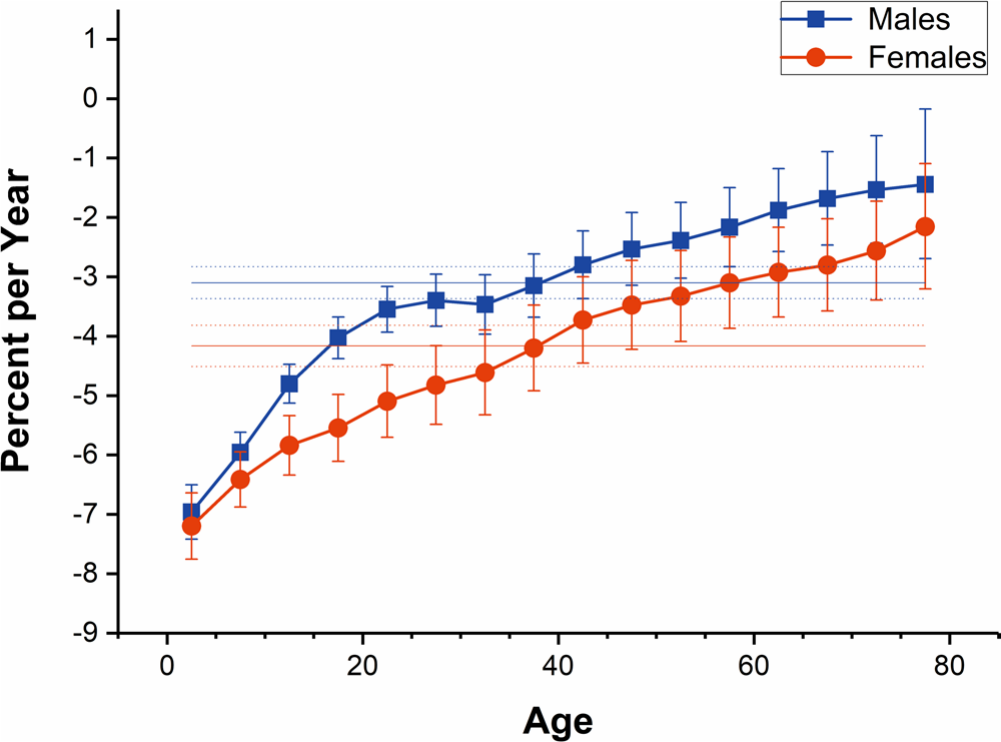
Local drift with net drift values for drowning mortality in China. Age group specific annual percent change (%) with the overall annual percent change (%) in drowning mortality rate and the corresponding 95% CI.

The net drift, which indicates the overall annual percentage change, and local drifts, which indicate annual percentage changes for each age group, were shown in Fig. 3. We found that the net drift was −3.10% (95% CI, −3.37% to −2.83%) per year for males and −4.16% (95% CI, −4.51% to −3.82%) per year for females. The local drift values were below 0 in all age groups in both males and females, which were lowest at ages 0–4 years (around −7.00% per year) and generally increased with age group in both sexes.

**Figure 4:**
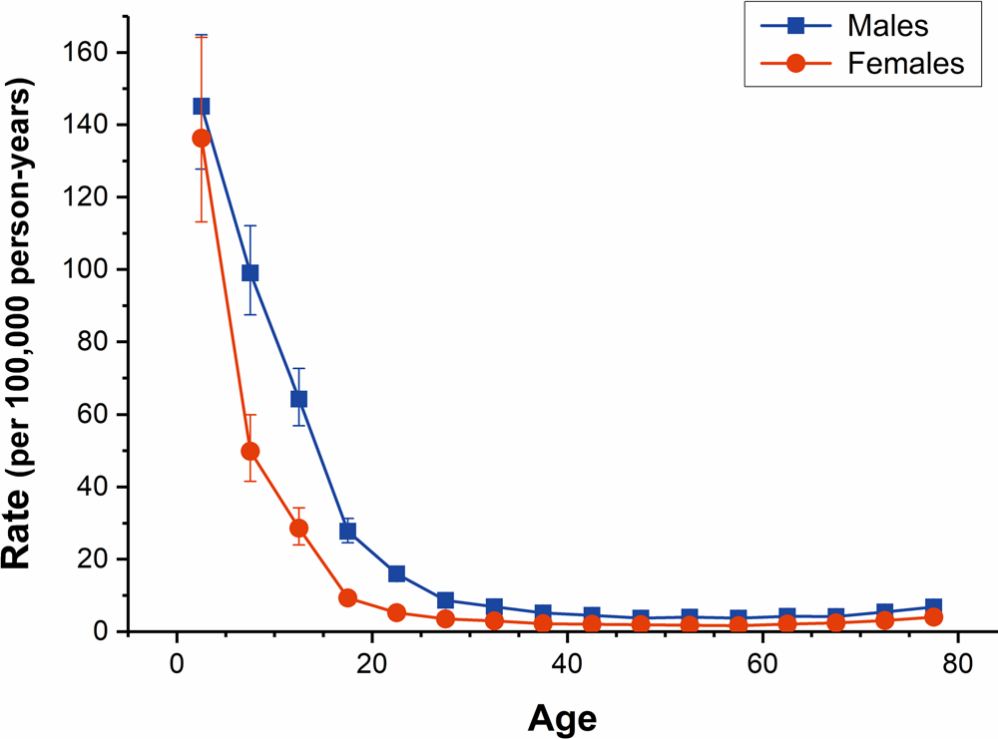
Longitudinal age curves of drowning mortality in China. Fitted longitudinal age-specific rates of drowning mortality (per 100,000 person-years) and the corresponding 95% CI.

The longitudinal age curves of drowning mortality in both sexes were displayed in Fig. 4 for both sexes, individuals in the same birth cohort witnessed a substantial decline followed by a slight increase in the risk of death from drowning with age. Specifically, the risk of death from drowning in both sexes decreased rapidly before 20 years old; this decreasing trend continued but decelerated thereafter; the risk rebounded marginally since the age of 50. Using the lowest risk of drowning death (the 55–59 age group) as a reference, the RRs of the top three risk group, i.e. the 0–4, 5–9, 10–14 age groups, were 39.06, 23.36, 15.41 respectively for males and were 82.08, 24.24, 11.84 respectively for females. Overall, the risk of drowning in Chinese males has been higher than that of females throughout the life phase.

**Figure 5:**
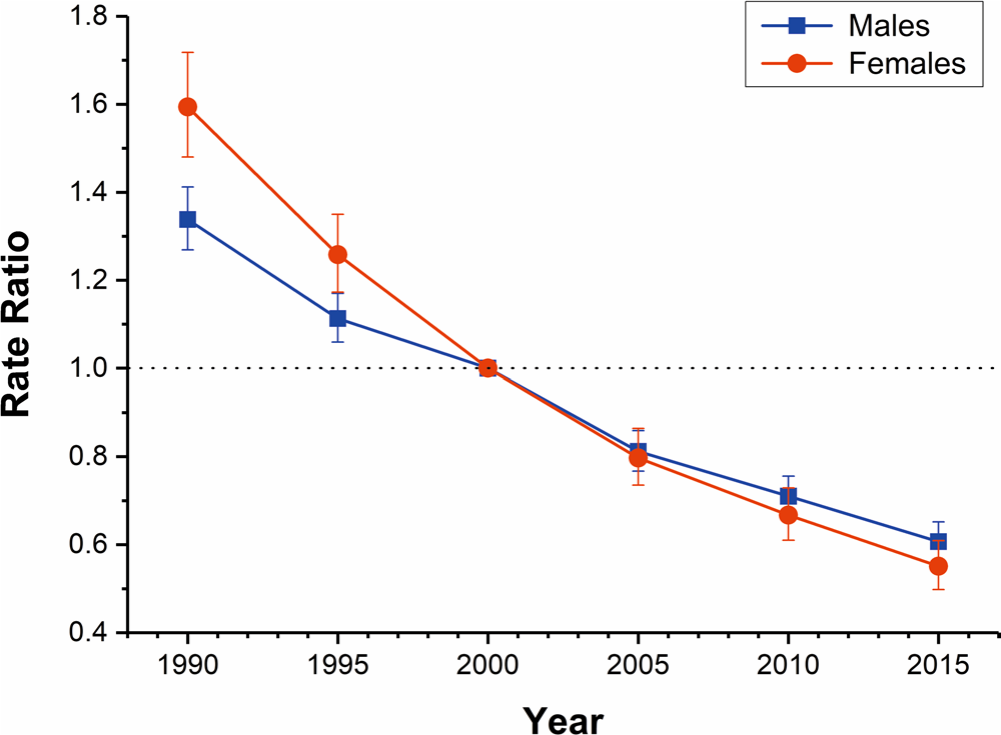
Period RRs of drowning mortality rate by sex in China. The relative risk of each period compared to the reference (year 2000) adjusted for age and non-linear cohort effects and the corresponding 95% CI.

**Figure 6:**
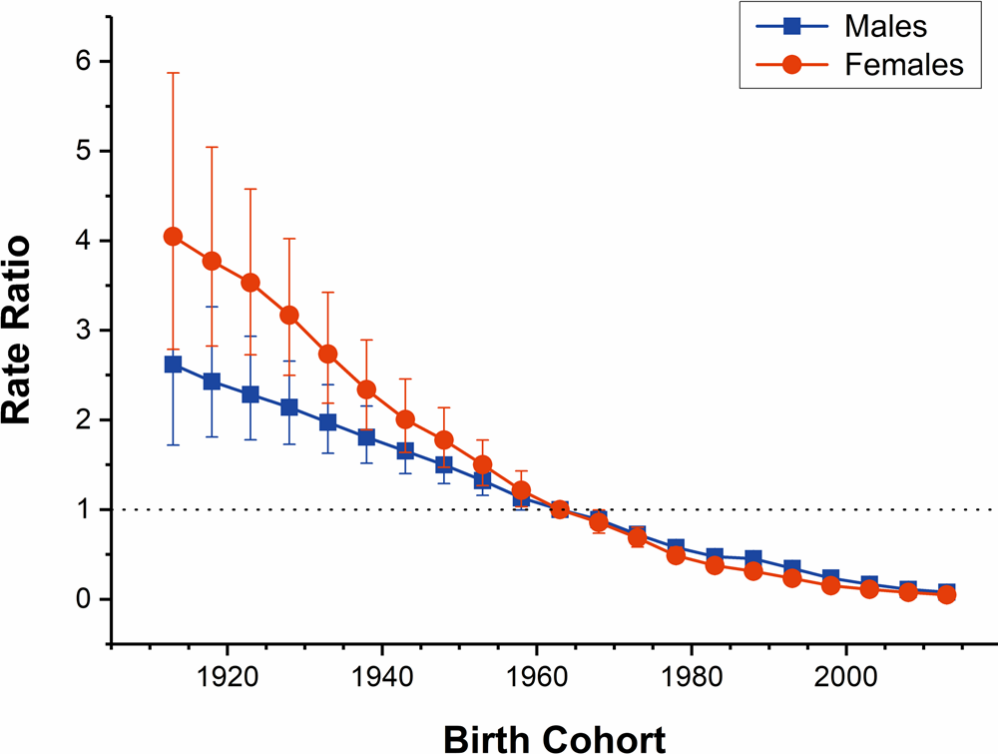
Cohort RRs of drowning mortality rate by sex in China. The relative risk of each cohort compared to the reference (cohort 1960–1964) adjusted for age and non-linear period effects and the corresponding 95% CI.

The estimated period and cohort RRs of drowning mortality in both sexes were displayed in Figs 5 and 6 respectively. The period RRs were found to have similar monotonic decreasing patterns for both sexes, with more reduction for females than for males during the whole study period. Compared with 1990, the RR of drowning in 2015 decreased by more than 50% (54.63% for males and 65.43% for females, respectively). Likewise, the cohort RRs were also found to display similar monotonic downward patterns for both sexes. Compared with the cohort 1911–1915, the RR of drowning of male and female in the cohort 2011–2015 was reduced by 97.02% and 98.81%, respectively. In addition, the results of the Wald tests demonstrated that there were statistically significant cohort and period RRs for both sexes (p < 0.01 for all), and so were the net drift and local drifts (p < 0.01 for all). It showed that the period deviations were smaller in magnitude than the cohort deviations, so the associations of period RRs with drowning mortality were mainly reflected by the net drift.

## Discussion

This is the first published study, to our knowledge, to investigate the long-term trends of gender-specific drowning mortality in China and to examine the age-, period-, and cohort-specific effects of them under the APC framework. Our results demonstrated that the ASMRs for drowning displayed general declining trends in both sexes with a decrease in the drowning mortality rate for every age group, and further suggested the existence of a cohort effect and a period effect for the drowning mortality trends.

Although drowning may occur to people of all ages, age is still one of the most important risk factors for drowning death. Using the latest data from GBD 2015, our results indicated that, the risk of death from drowning in the same birth cohort were both extremely high in the age groups among 0–19 years in Chinese male and female after adjusting for period deviations. This phenomenon is consistent with the internationally recognized awareness of the high-risk age groups of drowning death^17,18^. This is mainly due to the own characteristics of young people, especially children and teenagers, and the lack of supervision on them^19^. Children and teenagers are curious and motivated with high-frequency wide-range activities and a lack of self-safety awareness; their abilities of balance (for young children), risk response, and emergency preparedness were relatively not developed. Therefore, without effective adult supervision, fatal drownings caused by slip or other accidents could be easily triggered when children and teenagers were frolicking, bathing, swimming or doing other activities in or near the water. The longitudinal age curve results also showed that the risk of death from drowning slightly rebounded in the 59–79 years old life stage in both sexes. This should be mainly associated with the deterioration of body function in the elderly and having more free time after retirement. Studies have shown that^20,21^, inconvenience, dizziness and their suffering from diseases (such as epilepsy, Alzheimer’s disease, heart disease, etc.) are often considered as important causes for sudden, unexplained submersions in the elderly. In addition, the increased access to water due to having more free time after retirement would increase the chance of drowning for the elderly, thereby increasing the risk of drowning mortality.

With regards to the temporal trend for drowning mortality, we should not ignore the possible effect from the revision of International Classification of Diseases (ICD) code during the study period. But at present, there were no relevant studies to prove that changes from the ICD-9th to ICD-10th had a substantial influence on the analysis of temporal trends for drowning mortality^22,23^, so the underlying reason for decreasing trend of drowning mortality in China was likely to be other factors. Among them, China’s rapid urbanization process in recent decades was probably the most important one. According to the existing literature^24,25^, urbanization could affect the risk of drowning in many ways. First, the urbanization process would reduce people’s access to natural waters (ponds, streams, rivers, reservoirs, lakes, etc.) and agricultural water sources (aquaculture water sources, irrigation water sources), while close to waters is a very important risk factor for drowning (especially for rural areas) and the vast majority of drowning in China occurred in natural waters^26^. Second, the process of urbanization would change the scope of people’s activities (from outdoor to indoor) and the way of their entertainment (becoming more diversified); even if some people still enjoy swimming, having fun in swimming pools is much safer than in natural waters. Third, accelerating urbanization has been regarded as an essential instrument for economic development and lessening regional income disparity in the developing world^27^. While per capita income increases with the degree of urbanization^28^, drowning rates vary inversely with per capita income for all ages combined^29,30^. It has been reported that China’s urbanization rate rose from 26.4% in 1990 to 56.1% in 2015, which means that a large non-urban population had completed the transfer to the urban areas. So it can be inferred that the rapid urbanization played a positive role in the decreasing trend of drowning mortality in China.

The study on urbanization and the risk of drowning in different age groups showed that^24^, urbanization had a more pronounced effect on reducing the risk of drowning among adolescents and children than in other age groups. It is probably because important protective factors such as beliefs, skills, and knowledge which were necessary to prevent or deal with drowning, were more effective to spread in the urban context than in the rural. If adolescents and children experienced the process of urbanization earlier in their life stage, they would have better abilities on how to avoid drowning. Therefore, for drowning mortality in China, urbanization could play an important role in the cohort effect. In addition, the improvement of public education in different birth cohorts should also be considered. Relevant information indicated that^31^, after the founding of People’s Republic of China (year 1949), education in the birth cohort 1950s and 1960s had been greatly improved compared with those cohorts born in pre-1949, which benefited from the vigorous promotion of literacy and the positive development of basic education; after the reform and opening up (year 1978), with the popularity of compulsory education and the expansion of higher education, education in the birth cohort 1980s and 1990s reached a new level compared with the older generations. A higher level of education is generally regarded as a protective factor for drowning because people with better educational backgrounds often resort to more protective measures such as taking swimming and water safety training or conducting water activities in safe waters^24^; if the child’s mother had only received primary education, the risk of drowning was significantly increased relative to the child whose mother had a higher education level^32,33^. So there is no doubt that the continuous improvement of public education contributed to a protective effect of the cohort effect on the risk of drowning in China. By analyzing the cohort deviations, we found that the moderation in cohort RRs of drowning mortality started from the cohort 1965–1969 for both Chinese men and women. It may be related to that, compared with higher levels of education, basic education has a greater impact on reducing the risk of drowning death.

Alcohol use was identified by WHO as an important risk factor for drowning^1^. Since China’s per capita consumption of alcohol showed an upward trend year by year^34^, alcohol use may also be one of the factors affecting the risk of drowning, mainly in a negative way. It has been postulated that alcohol raises the risk of drowning by spoiling judgment and performance as well as through direct physiologic impacts that influence survival once a submersion happens^35^. In addition, many studies have shown that parents or guardians with drinking were also considered to be an important risk factor for drowning in children^21^. However, Chinese researchers are currently lacking awareness of these. Although the search for “drowning & drinking/alcohol” through the major Chinese web search engines would find tons of news reporting that a lot of drowning events were related to drinking, researches on drowning and alcohol use in China were almost blank to date. This is likely to be associated with the fact that the Chinese routine forensic examination of drowning does not include the blood alcohol content test^36^. Additional research is needed to confirm the role of alcohol use as a risk factor for drowning in China.

Our study has several limitations. First, it is evitable that the completeness and accuracy issues on drowning mortality data may somehow lead to bias in our study, although there were many adjustment steps in GBD 2015, including^37^ the corrections to incompleteness, under-reporting, and misclassification, as well as the redistribution of the garbage codes^2^, to enhance the data quality and comparability. But most would agree that the possible bias in the present study has been reduced a great deal, compared with that of research utilizing the unadjusted raw data. Second, since the original data for China’s cause of death is only provided by five-year age groups while the age and period intervals must all be fixed and equal in APC analysis, so this APC analysis had to be performed in periods of multiples of five years. It should be noted that certain subtle variations in age, period and cohort effects may have been smoothed out by grouping data into five-year intervals. The drowning mortality data should be analyzed with as small as possible units to detect those possible subtle variations when the smaller age group data could be accessed in the future. Third, similar to other APC investigations, there was the possibility of existence of ecological fallacy for the reason that interpretations from results at population levels do not necessarily hold for individuals^38^. Therefore, the related speculations brought up in our study still need further confirmation in the future individual-based investigations.

## Conclusions

In conclusion, our study demonstrated that the ASMRs for drowning in both sexes displayed general declining trends with a decrease in the drowning mortality rate for every age group, and in the same birth cohort, both sexes witnessed a substantial decline followed by a slight increase in the risk of death from drowning with age after controlling for period deviations. The estimated period and cohort relative risks were found in similar monotonic downward patterns for both sexes, with more reduction for females than for males during the whole study period. The patterns are likely to be related to China’s rapid urbanization process and the continuous improvement of public education. The role of alcohol use as a risk factor for drowning in China warrants further investigation.
